# Integrated Management of Residential Indoor Air Quality: A Call for Stakeholders in a Changing Climate

**DOI:** 10.3390/ijerph14121455

**Published:** 2017-11-25

**Authors:** Marie-Eve Levasseur, Patrick Poulin, Céline Campagna, Jean-Marc Leclerc

**Affiliations:** Environmental Health Department, Institut National de Santé Publique du Québec, 945, avenue Wolfe, 4e étage, Québec City, QC G1V 5B3, Canada; marie-eve.levasseur@inspq.qc.ca (M.-E.L.); celine.campagna@inspq.qc.ca (C.C.); jean.marc.leclerc@inspq.qc.ca (J.-M.L.)

**Keywords:** indoor air, health, indoor environment, climate change, adaptation, public health

## Abstract

A paradigm change in the management of environmental health issues has been observed in recent years: instead of managing specific risks individually, a holistic vision of environmental problems would assure sustainable solutions. However, concrete actions that could help translate these recommendations into interventions are lacking. This review presents the relevance of using an integrated indoor air quality management approach to ensure occupant health and comfort. At the nexus of three basic concepts (reducing contaminants at the source, improving ventilation, and, when relevant, purifying the indoor air), this approach can help maintain and improve indoor air quality and limit exposure to several contaminants. Its application is particularly relevant in a climate change context since the evolving outdoor conditions have to be taken into account during building construction and renovation. The measures presented through this approach target public health players, building managers, owners, occupants, and professionals involved in building design, construction, renovation, and maintenance. The findings of this review will help the various stakeholders initiate a strategic reflection on the importance of indoor air quality and climate change issues for existing and future buildings. Several new avenues and recommendations are presented to set the path for future research activities.

## 1. Introduction

Energy efficiency in residential buildings has become one of the central concerns in recent decades, and has resulted in more airtight constructions. To compensate for the decrease in natural ventilation, which used to be sufficient to ventilate buildings, mechanical ventilation, and other measures, such as air extractors and purifiers, have been developed to deliver good air quality to ensure occupant health and comfort. These new realities have led to an increase in indoor air quality (IAQ) research and interest. In addition, a paradigm change has been observed concerning the management of environmental health issues. As an example for IAQ, from managing specific problems individually, it now seems more appropriate to manage IAQ in a holistic manner and to consider several determinants of IAQ when implementing solutions. Even though it can still be relevant to resolve problems one contaminant at a time (e.g., repairing water infiltration in the foundations to eliminate water infiltration), it would be beneficial to consider other potential IAQ issues that could be tackled by similar measures (e.g., installing a radon mitigation system while sealing the foundations to prevent the introduction of water and underground gases). Although never concretely proposed, such an integrated approach to IAQ management should be considered by building managers, owners and occupants, as well as public health practitioners to improve IAQ and occupant health.

IAQ varies according to the building characteristics (e.g., condition and type of foundation, presence or absence of mechanical ventilation, airtightness, and envelope integrity), the occupants’ behaviors (e.g., ventilation habits, indoor smoking, and choice and use of cleaning products), and the characteristics of the outdoor environment (e.g., climatic conditions and physiographical context) [1]. Apprehending the indoor environment in its entirety entails taking into account all of the variables potentially affecting the indoor parameters, including the prevailing and future outdoor conditions. It is therefore particularly relevant to place this approach in a climate change (CC) context, since it can account for climatic variations and take into consideration several determinants of IAQ that will be modified by CC.

In fact, CC presents additional pressure on our societies by modifying the frequency, extent, and intensity of various climatic processes, including extreme weather events [2]. From a public health perspective, these phenomena can generate direct and indirect threats to the general population. Among these, increasing temperatures and the associated impacts can affect the IAQ of residential buildings. Some examples include temperature variations that can modify air conditioning and heating needs, the often associated increase in relative humidity that can create favorable conditions for the growth of certain microorganisms, and thawing permafrost that can affect building integrity. Moreover, variations in the frequency and quantity of precipitation can affect the built environment [3,4] (e.g., building damage from floods, ground movements, and mold development). An increased frequency of smog episodes and forest fires can also alter outdoor air quality and exacerbate infiltration of several contaminants [1,3]. Various measures have been proposed to mitigate the effects of CC on occupant health and comfort, for the general population, as well as for vulnerable groups (according to age, health and socioeconomic status, type, location, and condition of the dwelling, etc.) [1].

In 2011, the Institute of Medicine of the National Academies overviewed the impacts of CC on IAQ; however, the adaptation measures presented were rather generic [1]. At the time, there was no detailed inventory of the possible adaptation measures applicable to residential buildings concerning IAQ. Literature on the links between climate change, indoor air quality, and health is still emerging [4,5,6,7,8].

In this paper, we review the literature on the determinants of IAQ in a changing climate context and propose a management approach that is situated at the nexus of three basic concepts: (1) reducing contaminants at the source, (2) ensuring adequate ventilation in buildings, and (3) purifying indoor air if needed and depending on the prevailing environmental (outdoor) conditions. These concepts will be further analyzed in the following sections.

## 2. Materials and Methods

In 2016, our group reviewed adaptation measures that are associated with IAQ in a climate change context. Six concepts were combined and researched in OvidSP and ProQuest databases (indoor air; climate change; contaminants; legislation, guidelines, recommendations, normalization, certification; building materials and decoration products; and, purification techniques). Articles from 2000 and after, published in English and French, and corresponding to the review objectives were retained, after filtering titles and abstracts for relevance. Snowballing and grey literature search were also used to complete the review process. Articles had to be related to northern climates, such as Canada and Europe. 

Inspired by the results of this review [9], and in light of the paradigm change previously mentioned, our group wrap up the main results and promote an integrated IAQ management approach in the context of CC to ensure occupant health and comfort, based on expert judgments and experiences.

## 3. Results and Discussion

The results of the review are presented through the nexus of three key concepts that should be implemented together—and not as individual strategies—to improve IAQ management in a changing climate.

### 3.1. Concept 1: Reducing Contaminants at the Source

Managing IAQ through an integrated approach requires first and foremost reducing contaminants at the source, namely by limiting the infiltration and emission of contaminants indoors.

#### 3.1.1. Reducing Contaminants through Coercive Measures

Coercive measures include mandatory dispositions comprised in codes, laws, and regulations. For example, building codes dispositions regarding wall, foundation, and floor insulation, or the installation of a bathroom exhaust fan or a kitchen range hood are considered coercive measures that are meant to directly or indirectly reduce contaminants at the source.

Several countries have adopted legislation regarding various aspects of building construction and IAQ issues. France [10], China [11], Denmark [12], South Korea [13,14], Japan [15], and Canada [16,17] have adopted regulations affecting IAQ directly or indirectly. For instance, since 2013, France requires all of the construction materials and interior decoration products sold in France to be labelled with a standardized label presenting information on the emission of volatile organic compounds (VOC) (e.g., formaldehyde) in order to provide clear and complete information about the products that consumers buy and install in their buildings [10].

Although it is first carried out for energy efficiency purposes, optimizing the building envelope (insulation and airtightness) may be considered as a way to reduce contaminants at the source, since it limits the occupants’ exposure to physical stresses and contaminants from the outside. Such improvements offer a passive and energy-efficient solution to mitigate the effects that are associated with heat waves and cold spells, especially for vulnerable populations [18]. Moreover, these measures can improve the occupants’ thermal comfort by keeping the building warmer in winter and cooler in summer [4,19], therefore lowering the demand for heating and air conditioning, which consequently reduces building energy consumption [20,21]—relevant measures in a context of CC.

Installing a membrane beneath the foundation slab or on the crawl-space floor, adding an air vent in the attic or in crawl spaces, and ensuring that the clothes dryer has an exterior exhaust are other measures available to limit the introduction or facilitate the evacuation of excessive humidity, soil gases such as radon, and mold development inside a building [22,23,24]. Provisions regarding the installation of low-emissivity materials could also be included in building codes to contribute to the reduction of contaminants at the source.

#### 3.1.2. Reducing Contaminants through Incentive Measures

Incentive measures comprise voluntary programs and certifications of construction, decoration, and furnishing products and materials, as well as those concerning building or neighborhood construction and development. These certifications are generally oriented toward energy efficiency and sustainability criteria, but sometimes include IAQ considerations, such as the Leadership in Energy and Environmental Design program, or LEED [25], or the Living Building Challenge [26]. Since the main objective of these programs is often to reduce the ecological footprint that is associated with the construction and use of buildings in a sustainable development perspective, these measures can contribute indirectly to CC adaptation by promoting a greenhouse gas (GHG) reduction strategy.

Various programs and certifications offer an alternative to building owners and managers that are wishing to improve or maintain the IAQ of a building for the health of their users. Several programs, such as LEED, include requirements regarding the products and materials that re installed in the certified buildings (e.g., low-VOC materials and GREENGUARD labelled materials). However, a brief overview of programs that are available in North America, particularly in Canada, indicated few programs offering dispositions specific to IAQ, and most are optional.

Certifications concerning construction and renovation products and materials aim at limiting the emission of contaminants throughout their life-cycle (e.g., formaldehyde and other VOCs) [27]. Among these environmental labelling schemes, Ecologo© (UL Environment certifications, Northbrook, IL, USA), Green Seal© (Washington, DC, USA), GREENGUARD© (UL Environment certifications, Northbrook, IL, USA), FloorScore^®^ (Resilient Floor Covering Institute, LaGrange, CA, USA and SCS Global Services, Emeryville, CA, USA), and Green Label© (Carpet and Rug Institute Inc., Dalton, GA, USA) are labels that are used in several countries. The information on the composition of the certified products and materials can help construction professionals, designers, contractors, and suppliers, as well as consumers, to make enlightened choices with regard to the products they select and install (e.g., no-VOC paints) [28]. Moreover, a series of international standards has been developed concerning indoor air (ISO 16000) that addresses certain issues, such as mold, asbestos, and VOCs. Currently, even though no evaluation seems to have been conducted concerning the efficiency of these labelling schemes to significantly reduce the occupants’ exposure to indoor air contaminants, selecting less-emissive materials is still considered an incentive measure to reduce contaminants at the source. 

#### 3.1.3. Reducing Contaminants through Voluntary Measures

Voluntary measures are implemented directly by buildings’ occupants, owners, and managers, and are driven by the will to live in or operate buildings with better IAQ. Since numerous indoor air contaminants are generated by the occupants’ activities and behaviors, reducing the contaminants at the source begins by changing certain individual behaviors or habits. IAQ can be improved by refraining from smoking indoors, correctly using and storing potentially toxic household products, correctly using pest control products (e.g., insecticides and fungicides), and only when necessary, correctly using fuel-fired equipment, and reducing activities that generate excessive humidity [29,30]. Exterior exhaust fans and hoods (e.g., in the kitchen and bathroom) also serve as efficient measures for removing contaminants at the source, such as fine particles and humidity, and limit their dispersal when used adequately [24]. Using a portable dehumidifier in conjunction with these systems in damper locations of a building, generally in the basement, is an efficient measure to reduce relative humidity, which in turn limits the development of suitable conditions for the proliferation of associated indoor air contaminants [24,31].

Proper building envelope design and occupant behavior can limit the infiltration of contaminants from the outside, while regular maintenance and inspection of buildings and their components are voluntary measures that can prevent the deterioration of building materials that are not designed to be exposed to the elements. Among these, synthetic materials, such as vinyl, need attention, since they can be degraded by the sun, wind, and rain, as well as by increasingly frequent freeze-thaw cycles that are caused by milder winter weather [22,23]. Even though governmental renovation programs are sometimes offered to building owners (e.g., *RénoClimat* program and *RénoVert* tax credit in Quebec, Canada, or the tax refund *Crédit d’impôt pour la Transition énergétique* and *Habiter Mieux* programs in France), they should consider inspecting and renovating their properties regularly, not only as an investment, but also as a way to improve IAQ and comfort, and to adapt their assets to anticipated climatic changes. 

### 3.2. Concept 2: Improving Ventilation in Buildings

Improvements in building energy efficiency are driven by the will to reduce energy costs and carbon emissions and to improve the occupants’ comfort. However, this reality comes with increased airtightness of building envelopes, therefore allowing for little exchange between indoor and outdoor air. Without proper ventilation, the contaminants generated inside are trapped in the building and contribute to the degradation of IAQ [1,32,33]. Since it is impossible to completely eliminate all of the contaminants at the source, and considering the gradual reduction in passive natural ventilation over the years, complementary ventilation measures are now necessary to improve and maintain IAQ in an integrated management approach. 

#### 3.2.1. Improving Ventilation through Recent Coercive Measures: Centralized Mechanical Ventilation in New Dwellings

To compensate for the increase in building air tightness, centralized mechanical ventilation systems were designed and installed in various building types in Canada since the 1980s. Their presence is now so important that the installation of mechanical ventilation has been mandatory since 2012 in every new building that is constructed in Quebec, Canada [34]. The most common systems in today’s buildings are those that are equipped with a heat or energy recovery module (HRV and ERV), coupled or not with a forced-air heating system [35,36]. A balanced calibration of these systems prevents pressurization, which allows for humid air to enter the walls, and depressurization of the envelope, which allows for the infiltration of humidity and outdoor gases, such as radon [37]. These systems ensure exchanges between indoor and outdoor air and its distribution throughout occupied spaces.

Most countries have adopted requirements regarding the total minimal capacity of mechanical ventilation in their building codes [38,39]. In most countries in Europe, the minimal ventilation rate for new buildings that are equipped with mechanical ventilation is around 0.5 air changes per hour (ACH), while in North America, the prescribed rate is between 0.30 and 0.35 ACH (corresponding approximately to the sum of natural and mechanical ventilation) [38,40,41]. Centralized mechanical ventilation and the associated air exchange rates are intended to dilute indoor contaminants, limit the introduction of outdoor contaminants, reduce heat losses, and ensure thermal comfort. According to several organizations, when installed, used, and maintained adequately, centralized ventilation systems, such as HRVs and ERVs make up some of the most important measures for maintaining IAQ in buildings [42,43,44,45,46].

These systems can also mitigate the impacts that CC has on certain comfort parameters, such as temperature and relative humidity [47]. For example, they are useful for maintaining relative humidity at acceptable levels and prevent the development of associated microorganisms [48,49]. However, these devices and their components must be maintained and cleaned properly to deliver their optimum performance and hinder them from accumulating and releasing other contaminants in the indoor air, such as inhalable particles that are likely to cause respiratory problems for the occupants [37,40].

#### 3.2.2. Improving Ventilation through Voluntary Measures: Natural Ventilation

Natural ventilation occurs through air infiltration in various openings and cracks in the building envelope (e.g., windows, doors, ventilation ducts, and edges of electrical outlets and switches) [50]. Passive natural ventilation takes place continuously in buildings at various degrees, depending on the pressure differences that are caused by the stack effect or by the wind effect [44].

Structural components (e.g., windows and doors) and voluntary occupant behaviors (e.g., opening windows to ventilate the dwelling) can increase the natural ventilation of a building. However, voluntary natural ventilation increases the introduction of outdoor contaminants (e.g., pollens, fine particles, and ozone), some of which can be amplified by CC (e.g., increased pollen concentrations with the extension of the pollen season) [3]. Controlling natural ventilation according to climatic variations and outdoor conditions, when possible, is an affordable measure that is accessible to almost everyone, even though its efficiency depends on several factors that make it difficult to control [44,51]. The necessary efforts from the occupants to manually open windows and control the natural ventilation [52] and their tendency to open windows only when perceiving a problem with IAQ or comfort [53] are among the factors that are affecting the efficiency of natural ventilation. Moreover, when it comes to windows, studies have shown that occupants are ambivalent when it comes to saving energy (i.e., reducing heat losses during winter and preserving coolness in summer) or maintaining good IAQ [51,54]. Other factors can compromise the use of natural ventilation, such as non-functional or unsuitable windows or doors, age of the occupants, unpleasant outdoor odors, feeling of insecurity, noise, and smog [24,44,52,55].

It is preferable to use voluntary natural ventilation at night, when outdoor air contaminants generally occur in lower concentrations [37]. Its efficiency largely depends on the difference between indoor and outdoor temperatures, on the thermal inertia of the building and the heat transfer capacity between the building massive components (e.g., concrete, brick, and roof shingles), and the flow of fresh air [18,54].

Finally, natural ventilation options are promoted in the American Society of Heating, Refrigerating and Air-Conditioning Engineers (ASHRAE)’s new residential ventilation standard—ASHRAE 62.2—(without identifying them precisely), but efforts in terms of design, performance evaluation, and certification will be needed from the industry in order to develop this alternative ventilation method [40].

#### 3.2.3. Improving Ventilation through Voluntary Measures: Optimizing Mechanical Ventilation

Optimizing existing ventilation systems may be an effective means to counteract the adverse effects of CCs [56]. Ventilation rates can be modulated in time and space according to indoor and outdoor conditions and the occupants’ needs. For example, by adequately controlling the existing systems, the ventilation rate of occupied spaces can be increased to meet the needs of the occupants, by adjusting the fan speed or the exchange-cycle duration if necessary, and then decreasing it as the situation returns to normal for energy-saving purposes [36]. It is also possible to control the ventilation system rates according to the indoor air relative humidity by attempting to respect the range that is recommended by most recognized organizations (such as US EPA (United States Environmental Protection Agency, Washington, DC, USA), ASHRAE (Atlanta, GA, USA), Health Canada (Ottawa, ON, Canada) and Canada Mortgage and Housing Corporation (Ottawa, ON, Canada)): between 30% and 50%, approximately [57]. To maintain relative humidity in this range, the use of a portable dehumidifier in damp building areas (e.g., basement) can be an effective solution, while also reducing the associated indoor air contaminants, such as mites or mold spores [24,31]. The choice of a low-energy model, which is adapted to the size of the building or the space, must be considered.

During events of high atmospheric pollution, such as forest fires or smog episodes, reducing the duration of the exchange cycle or temporarily interrupting all mechanical ventilation may limit occupant exposure to outdoor contaminants [58,59]. In this case, the building acts as a shelter and as a fresh air tank. This temporary confinement measure can only be effective if doors, windows, and exhaust fans are kept closed. In addition, the more airtight the building is, the more effective this measure will be.

Mechanical ventilation can even be adjusted in real time according to occupants’ needs and indoor conditions when equipped with an intelligent control system [60,61,62,63]. The use of intelligent controls and appropriate probes allow for a simultaneous monitoring of many variables, such as occupancy, temperature, relative humidity, and CO_2_ concentration in indoor air. The collected data are computed by an interface device that provides flexible and optimal ventilation of occupied spaces, while minimizing the operating costs that are associated with heating and air conditioning. Although these systems are not commonly encountered in residential buildings, this indoor air management approach could become popular over the coming years, especially because of its energy saving potential [64,65]. If outdoor conditions, such as wind, humidity, temperature, and outdoor contaminant concentrations are also monitored in order to adjust the mechanical ventilation flow rate, this measure could become even more relevant for adapting to CC.

Finally, despite the demonstrated effectiveness of residential ventilation systems in improving IAQ, consumers should base their choice on the overall costs (i.e., purchase, installation, and maintenance), as well as energy consumption and the desired control options.

### 3.3. Concept 3: Complementary Measures to Maintain Indoor Air Quality and Comfort

#### 3.3.1. Maintaining Indoor Air Quality through Air Cleaning Devices

Despite the efficiency of the measures outlined above, some contaminants may persist in indoor air as a result of uncontrollable pollution sources (e.g., highways, mines, and factories). In addition, some people are more vulnerable to indoor air contaminants, such as individuals that are suffering from asthma or allergies who may react to low levels of contaminants. In these circumstances, the use of an indoor air cleaning device could be a complementary measure to improve IAQ in certain rooms (e.g., bedroom or living room).

Various indoor air cleaning processes are available to consumers that are wishing to reduce their exposure to particulate and gaseous contaminants [36]. These purification devices can be integrated into specific centralized ventilation systems, while others can be deployed as portable units. In the first case, a centralized ventilation system equipped with an external air filtration device can prevent the introduction of outdoor pollutants. The pore diameter of the filter must be adapted to the load capacity of the fan and the nature of the contaminants to be considered. While high efficiency particulate air (HEPA) filters are very efficient (filtering 99.97% of particles less than 0.3 μm), their replacement cost can be prohibitive. The introduction of a new generation of electronic filters could be promising, since they combine filtration efficiency and a low cost of use and maintenance. Their effectiveness, however, depends on several factors, including the rigorous maintenance of filters [66].

In the second case, portable treatment devices must be installed at specific locations, usually in the vicinity of contaminant sources or near the vulnerable individuals. Despite their theoretical performance, the effectiveness of air cleaners varies greatly, depending on their design, installation, use, and maintenance, as well as the nature and concentration of contaminants that are present in indoor air [59,67,68,69]. Moreover, the use of this type of appliance should be considered as an alternative or a complementary solution to mechanical or natural ventilation when indoor or outdoor air quality is altered—situations that could occur more frequently in a CC context [37,59,67,70,71].

Examples of commercially available air cleaning devices include devices controlling particle concentrations (e.g., dust, mold spores, pollens, and mites), gases (e.g., formaldehyde, ozone, NO_x_, and SO_2_), or both. Certain devices incorporate an activated carbon cartridge or other chemical adsorbents, an electronic precipitator, or a photocatalytic oxidation cell. Although these devices have well-documented theoretical efficiency, few experiments have been carried out under real conditions or to account for future climate scenarios. In addition, the costs of acquiring, using, and maintaining these devices can be prohibitive for consumers in the housing sector. For example, the effectiveness of activated carbon is known to be relatively short and remains variable in different contexts [59,72,73]. Electrostatic precipitators require rigorous maintenance and are likely to emit ozone, and are therefore not recommended in residential settings [67,71,74], while photocatalytic oxidation systems may emit certain VOCs [71,75,76], as well as NO_2_ [77]. In certain concentrations, these contaminants can affect the occupants’ health.

#### 3.3.2. Maintaining Indoor Air Quality and Comfort through Air Conditioning

Air conditioning is among the main issues that building specialists will have to face in the context of CC [18]. Although it is still the most effective measure for reducing morbidity and mortality among vulnerable people during extreme or prolonged heat events [78], inadequate use of air conditioning may contribute to reduced physiological adaptation to heat [79]. Inactive individuals who spend most of their time in fresh indoor environments limit their thermoregulatory capacity (or adaptation) in warmer temperatures [80]. They are more likely to suffer from heat, since their vulnerability is greater than individuals who are physiologically adapted to heat, even when exposed to moderately but prolonged warm temperatures.

Additionally, air conditioning in urban areas contributes to the urban heat island phenomenon, increases electricity consumption, and the associated risks of power failure—especially during periods of high demand [4,79], and is associated with GHG emissions [19,45,81,82,83,84,85,86]. For example, the widespread use of air conditioners at the global scale could annually generate about 23 billion tonnes of GHGs by the end of the century [81]. 

To remedy these problems, passive control measures are recommended to reduce the risks of overheating and the need for air conditioning [4]. Among these, solar-shading devices are highly effective for reducing overheating, but it would seem the most effective interventions combine several adaptation measures (e.g., insulation, cool roofs, and shading devices).

## 4. The Nexus: Managing IAQ through an Integrated Approach in a Changing Climate

Managing IAQ through an integrated approach entails taking into account the determinants of IAQ and the anticipated impacts of CC, from building conception to the choice of materials and furnishings, and includes adequate ventilation and adapted occupant behaviors. By reducing contaminants at the source, improving ventilation, and, when necessary, purifying or modifying the indoor environment, building managers, designers, and occupants can improve IAQ and comfort, reduce concentrations of several contaminants, and orient occupants toward energy-efficient and healthy measures to maintain IAQ.

This strategy has been recommended, sometimes informally, and used in a number of construction or renovation projects. It also seems to be embedded in an increasing number of ecologically responsible construction programs. Most of them focus on sustainable development and energy efficiency principles (e.g., LEED^®^ (Leadership in Energy and Environmental Design, US Green Building Council, Washington, DC, USA), Net Zero Energy Housing© (Net Zero Energy Housing Council, Canadian Home Builders' Association, Ottawa, ON, Canada), and Passivhaus© (Passive House Institute, Darmstadt, Germany)), although some programs include IAQ and health concerns in their certification process.

In addition to measures that are applicable indoors, an integrated IAQ management approach should include measures pertaining to the outdoor environment. Although this review does not specifically cover them, measures such as the creation of outdoor cool areas or parks, reduction of atmospheric pollution, and sustainable stormwater management in urban centers can directly or indirectly influence IAQ by limiting outdoor pollutant infiltration and by maintaining control over comfort parameters. Such measures are generally inexpensive and are relatively easy to implement [19]. Moreover, these adaptation measures often offer co-benefits by reducing the impacts of CC.

## 5. Recommendations for Stakeholders

On the basis of this review and based on our expert judgment and experience, we were able to draw general recommendations for different stakeholders. In addition to legislation improvement and the development of programs to improve IAQ and energy efficiency in buildings, stakeholders and concerned authorities should take more initiatives and develop integrated adaptation plans for building owners and managers in order to prevent and mitigate the expected impacts of CC on thermal comfort and IAQ [87].

- **State/Provincial/regional authorities**: Since IAQ can be indirectly affected by outdoor air quality, urban development plans should consider the health impacts of the environmental measures implemented. For example, the reduction of atmospheric pollution and urban heat islands can be achieved by providing means of active transportation, establishing a mandatory canopy index, or efficiently managing stormwater. In addition to the mandatory installation of centralized mechanical ventilation systems in new buildings, the relevant authorities should consider requiring inspection and maintenance plans for these systems, and for the building envelope when granting construction permits in their territory. The adoption of regulations or even a renovation code incorporating the most pertinent measures should also be considered.
- **State/Provincial/regional authorities**: Governmental authorities should also promote the selection of adaptation measures offering multiple co-benefits in their decisions and policies in order to maximize their impact. For example, improving insulation and airtightness can reduce the building’s energy demand and prevent infiltration of contaminants from outside or from the soil (e.g., fine particles, nitrogen dioxide, carbon monoxide, ozone, moisture, and radon). Measures contributing to the reduction of GHG emissions (e.g., construction and renovation of more sustainable buildings that consume less energy) are also preferred, since these gases are one of the main causes of CC.
- **Managers of public housing and buildings**: The design, construction, and renovation of buildings hosting vulnerable and sensitive individuals, such as hospitals, daycares, senior residences, and subsidized housing, should be carried out using an integrated approach, i.e., by considering all of the measures that can promote IAQ and comfort and that take adaptation to CC into account.
- **Public health authorities**: An integrated management approach should be adopted in health establishments and in building renovation and construction processes (e.g., for hospitals) to account for CC and IAQ concerns. Public health authorities should also advocate to stakeholders for the inclusion of these issues in all types of buildings, in a health protection perspective.
- **Public health authorities**: Public health authorities should also inform and educate the general population about the appropriate behaviors to adopt for improving and maintaining IAQ in their homes in the context of CC [30,36]. Efficient and energy-saving measures should also be promoted to protect the most vulnerable populations, as well as to support social equity and accessibility.
- **Construction and renovation industry**: Raising awareness among construction and renovation stakeholders regarding the health impacts of emissions from construction products and materials should be continued, as well as promoting the use of less emissive products in buildings [30,36].
- **Construction and renovation industry**: Buildings should be designed or renovated to take into account their impacts on health and comfort, and should address the impacts of CC. Passive measures, such as solar-shading devices, should be favored to maintain a comfortable indoor environment and reduce the need for energy that is associated with air conditioning and heat adaptation measures [4].

## 6. Conclusions

When approaching IAQ problems, efforts should be oriented on finding solutions that take into account several parameters and can therefore be useful to solve or avoid multiple issues. Attention should be given to the importance of reducing contaminants at the source (including the choice of non-emissive materials, increasing air tightness, and insulation), improving ventilation and, when relevant, purifying or treating the indoor environment. The integrated IAQ management approach that is presented here should be considered by decision makers, managers, public health players, and occupants that are wishing to maintain good IAQ in the context of CC.

Although there has been important knowledge development and promising technological improvements in the field of IAQ in recent years, several areas of research have yet to be explored. Architects and engineers must keep designing and building performant buildings (e.g., green, sustainable, ecological, and net-zero energy) that integrate not only IAQ and health concerns, but energy efficiency and sustainability as well. Certification schemes for materials and products and for buildings should help to achieve certain standards of energy efficiency and IAQ in line with the current climate and that to come.

Chemical compounds that are emitted from construction and decoration products and materials should be better characterized, and new standardized methods should be developed for that purpose, with particular attention to current and anticipated climatic conditions. Additional information on building microbiomes should also be obtained in light of actual and future environmental conditions.

Research could also target the feasibility of developing a universal labelling scheme to categorize products according to their emissions. Consumers and promoters could then make informed choices about the materials that they select and install in their buildings.

Additional research is also needed to assess the efficiency of various components of mechanical ventilation systems, such as easy-to-use control modules. These findings could later be incorporated into building codes if they are proven to be effective. Further studies should be conducted to compare the efficiency of different indoor filtration devices under specific atmospheric conditions (e.g., during smog episodes) and validated climate scenarios. Finally, the anticipated increase in temperatures over the next decades highlights the need to further study the adaptation of thermoregulatory mechanisms, especially for vulnerable individuals.
